# Editorial: Substrate-enzyme interactions in lignocellulosic biodegradation

**DOI:** 10.3389/fbioe.2024.1503104

**Published:** 2024-11-19

**Authors:** Yunzi Hu, Wenlong Xiong, Nattha Pensupa, Chenyu Du

**Affiliations:** ^1^ Department of Bioscience and Biotechnology, College of Life Science and Technology, Jinan University, Guangzhou, China; ^2^ State Key Laboratory of Cotton Bio-breeding and Integrated Utilization, School of Chemical Engineering, Zhengzhou University, Zhengzhou, China; ^3^ School of Agriculture Natural Resources and Environment, Naresuan University, Phitsanulok, Thailand; ^4^ School of Applied Science, University of Huddersfield, Huddersfield, United Kingdom

**Keywords:** lignocellulose, biorefinery, cellulase, pretreatment, fermentation, saccharification

Application of enzymes to facilitate lignocellulose biorefinery has long been recognized as the most viable strategy to produce cellulosic biofuels, bio-materials and bio-chemicals. Globally, various research institutes and companies, such as DuPont and DSM, have established numerous pilot and industrial-scale production systems for bioconversion processes. However, few of them have been able to run commercially, due to the poor economic perspective. The key to a sustainable bio-production is high enzymatic degradation efficiency of lignocellulosic biomass to fermentable sugars, which depends on an appropriate pretreatment, excellent enzyme activities and well-matched interactions between particular substrate and enzyme synergistic actions. The reaction system is of great complexity that involves multiple macro- and micro-factors of substrate properties, diverse enzymes and intervention from other proteins. Numerous studies have been devoted to this subject for many years and achieved great progress that deserves to be noticed. In this context, research articles and critical reviews were collected in this Research Topic.

Breaking the complex bonding structure between the three components, cellulose, hemicellulose, and lignin by pretreatment technology highly contributes to improving the degradation efficiency of lignocellulosic biomass. Liu et al. had combined sulfomethylation and Fenton oxidation reaction to pretreat the bamboo, towards high efficiency and low-cost. This method fully exposed cellulose by increasing the porosity and delignification, as well as reducing the crystallinity of cellulose. In consequence, the efficiency of enzymatic hydrolysis of bamboo and the subsequent ethanol production was significantly improved, demonstrating a good investigation on promoting the feasibility of lignocellulosic biorefinery.

Wood-decaying fungi play an extremely important role in maintaining ecosystem health through accelerating lignocellulose degradation. By whole genome sequencing and annotating, Ma et al. had found that *Daedaleopsis sinensis*, a wood-decaying fungus, could produce abundant carbohydrate-active enzymes, which indicated that *D. sinensis* possessed not only strong ability to degrade cellulose but also potential ability to degrade hemicellulose, lignin, and pectin. This provides a new promising strain for lignocellulosic biodegradation.

The understanding of transcriptional network of enzyme producing strains is crucial for enhancing cellulase production. Siebecker et al. investigate transcriptomic factors of an industrially used strain, *Thermothelomyces thermophilus*. Cell growth, protein secretion, and transcriptomic profiles of strains lacking the cellulolytic regulators Clr1, Clr2, and Clr4 were studied. The *clr1* and *clr2* deletion strains failed to grow on cellulose, indicating their essential role in cellulose catabolism, with reduced expression of cellulase and related genes. In contrast, the *clr4* deletion strain showed similar growth to the parental strain but had increased expression of cellulases, hemicellulases, pectinases and esterases. The findings suggest that Clr1 and Clr2 activate the expression of these genes, with Clr1 controlling basal cellulase expression and initiating responses to cellulose, while Clr4 appears to repress this response. The potential new regulators involved in carbohydrate catabolism and enzyme expression were identified by comparative transcriptomics for further study in this area.

As the key barrier of cellulase hydrolysis, lignin is the most difficult component to degrade that requires an extracellular oxidative multi-enzymatic system. Aryl-alcohol oxidases are key members of this system. By combining steady-state and transient-state kinetics, turnover studies and isothermal titration calorimetry, Serrano et al. revealed that the activity of aryl-alcohol oxidase from *Bjerkandera adusta* was limited by the reoxidation of the Flavin; while the one from *Pleurotus eryngii* was limited by reductive half-reaction. The dehydrogenase activity of aryl-alcohol oxidase from these two different sources were limited by the hydroquinone release from the active site. Unveiling the kinetic characteristics of enzymatic catalysis in the fungal decay of lignocellulose could definitely contribute to design more advanced lignocellulosic degradation enzyme preparations.

Saccharification is to convert polysaccharides of lignocellulosic biomass to sugars, which are then converted to ethanol or other bio-based fermentation products. The study of Tang et al. revealed that incorporating lytic polysaccharide monooxygenases (LPMOs) could improve the saccharification efficacy of commercial enzyme preparations under proper condition. An appropriate aeration in hybrid hydrolysis and fermentation increased the conversion efficiency of glucan. It was because the gas flow could lead to evaporation of inhibitors derived from pretreatment process, such as heteroaromatic aldehydes (e.g., furfural), aromatic aldehydes, and an aromatic ketone (acetovanillone). This indicated that further research is needed to fully exploit the advantages of LPMOs and other auxiliary enzymes towards industrial application.

In summary, expanding knowledge and emerging technologies have indeed promoted enzymatic degradation efficiency of lignocellulosic biomass. However, establishing an appropriate method to evaluate and integrate different technologies is still challenging. Beyond laboratory tests, techno-economic analysis based on demonstrations carried out in pilot and large-scale production is necessary to fill in the gap between academic research and industrial application.

